# 18F-FDG-PET/CT can be used to predict distant metastasis in hypopharyngeal squamous cell carcinoma

**DOI:** 10.1186/s40463-022-00568-8

**Published:** 2022-04-01

**Authors:** Shinsuke Suzuki, Satoshi Toyoma, Tomoe Abe, Tentaro Endo, Teppei Kouga, Yohei Kaswasaki, Takechiyo Yamada

**Affiliations:** Department of Otorhinolaryngology and Head and Neck Surgery, Akita University Graduate School of Medicine, Akita, 010-8543 Japan

**Keywords:** Hypopharyngeal squamous cell carcinoma, Distant metastasis, 18F-FDG-PET/CT, Maximum standardized uptake value, Survival

## Abstract

**Background:**

Hypopharyngeal squamous cell carcinoma (HPSCC) has a high rate of distant metastasis, resulting in poor prognosis. The role of the maximum standardized uptake value (SUVmax), which was assessed via pretreatment 18-fluorodeoxyglucose positron emission tomography (FDG-PET), and computed tomography (CT) was examined, for predicting distant metastasis and survival.

**Methods:**

This study included 121 patients who underwent pretreatment FDG-PET/CT scanning and subsequent treatment for HPSCC. The SUVmax was measured via FDG-PET/CT. A receiver operating characteristic (ROC) curve analysis was used to determine whether the SUVmax was a predictor of distant metastasis and to select the best cutoff value. Univariate and multivariate Cox hazard regression analyses were used in identifying associations between the SUVmax and other clinicopathological factors with distant metastasis-free survival.

**Results:**

Distant metastases were identified in 33 patients during the median follow-up of 24 months after treatment. The ROC curve analysis determined that SUVmax was predictive of distant metastasis and identified a SUVmax of 13.9 as the best potential cutoff value. The univariate analysis showed that T and N classification, clinical stage, and SUVmax were significantly related to distant metastasis. However, in multivariate analysis, an SUVmax ≥ 13.9 was the only independent predictor of distant metastasis. Patients with high SUVmax values displayed significantly shorter distant metastasis-free survival and overall survival.

**Conclusions:**

SUVmax determined via pretreatment FDG-PET/CT is useful for predicting distant metastasis, distant metastasis-free survival, and overall survival in patients with HPSCC.

**Graphical Abstract:**

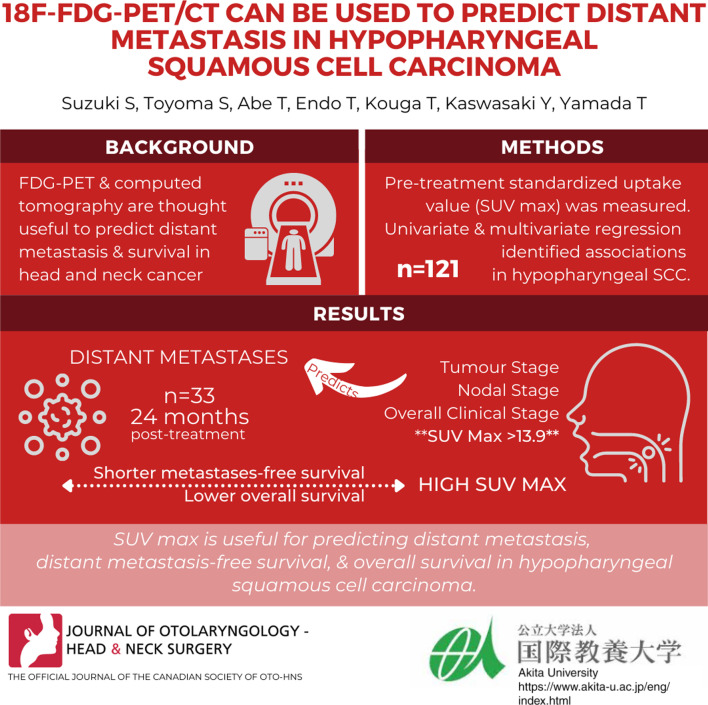

## Background

Hypopharyngeal squamous cell carcinoma (HPSCC) is one of the most common head and neck cancer types. Multidisciplinary treatment is administered following the disease stage, but the prognosis is poor because of recurrence and metastasis [1, 2]. Distant metastasis is a significant prognostic factor, and its prediction affects treatment decisions [3]. Therefore, several attempts have been made to better predict distant metastases [4].

Recently, 18-fluorodeoxyglucose positron emission tomography (FDG-PET) and computed tomography (CT) have been reported as in predicting prognosis in malignant tumors, including those of the head and neck [5]. Various types of metabolic parameters can be derived from FDG-PET/CT. Among these, the maximum standardized uptake value (SUVmax) has gained much attention and has been examined as a prognostic factor in head and neck cancer [6, 7].

For HPSCC, pretreatment SUVmax has been reported as a predictor of overall survival, prognosis after salvage treatment in recurrent cases, and larynx preservation after chemoradiotherapy [8, 9]. However, few reports have focused on the significance of pretreatment SUVmax in predicting HPSCC distant metastasis [10], and further studies are required to improve its accuracy.

Therefore, the aim of this study was to investigate the utility of pretreatment FDG-PET/CT SUVmax as a prognostic factor, including its ability to predict distant metastases in patients with HPSCC.

## Materials and methods

### Patients

Patients who were newly diagnosed (not included patients with recurrent disease or patients who have been previously treated with HPSCC) with HPSCC had available pretherapeutic whole-body FDG-PET/CT images and received treatment with a curative intent between January 2010 and December 2018 at the Department of Otorhinolaryngology–Head and Neck Surgery of the Akita University Hospital (Akita, Japan), and were retrospectively assessed. Patients with distant metastasis before therapy (n = 6), patients who did not receive complete treatment because of poor general condition (n = 3), and patients with insufficient FDG-PET/CT data for the quantitative analyses (n = 29) were excluded. Ultimately, 121 eligible patients were included in the final analysis.

This study was conducted under the review and approval of our Institutional Review Board. Given the study's retrospective nature, informed consent from each patient was waived.

### Staging and treatment

The patients were staged following the Union Internationale Contre le Cancer (UICC), TNM staging for head and neck cancer, 8th edition, 2017 [11]. The 121 patients were divided into two groups according to their primary tumor treatment modality: the first group included patients who underwent curative surgery as the first-line treatment (surgery group; n = 59). These patients were treated with postoperative radiation therapy (RT) with or without chemotherapy if they were at high risk (positive margins or less than 5-mm from the margin and/or multiple neck lymph node metastases). The second group included patients treated with radical RT with or without chemotherapy (RT group; n = 62). The primary treatment modality selection depended on whether the patients desired larynx preservation. Patients with T1 disease without lesions or distant metastases underwent trans-oral tumor resection. The patients in the RT group were treated with conventional radical RT with a total dose of 60–70 Gy, with 1.8–2 Gy per fraction; all other RT procedures were conducted as previously described [12, 13]. In the RT group, 60 patients received concomitant chemotherapy, including 34 patients with docetaxel-based chemotherapy, 24 patients with cisplatin-based chemotherapy, and two patients with cetuximab-based chemotherapy; two patients were treated with RT alone due to a poor general condition. Five patients received planned neck dissection.

Following treatment, patients with early locoregional recurrence (LR) were identified at an outpatient clinic, and salvage therapy was conducted after treatment completion. Table 1 shows the clinical characteristics of all patients.

**Table 1:** Characteristics of study patients (No. of patients = 121)

Characteristic	Number of patients
Age, y, median (IQR)	67 (61–73)
Sex, male/female	113/8
*Site of primary tumors at initial presentation*	
Pyriform sinus/postcricoid/posterior wall	88/12/20
SUVmax primary tumor	14.4 (11.8–19.7)
*Recurrence/metastasis*	
Locoregional alone	28
Local + Distant	3
Distant metastasis	31
Time to distant metastasis, mo, median (IQR)	24 (9–43)
*TN and clinical stage*	
T1/T2/T3/T4	8/50/27/36
N0/N1/N2/N3	29/10/78/4
Stage I / II / III / IV	6/17/12/86
*Primary treatment*	
Sugery/(C)RT	59/62
*Follow‐up information after recurrence*	
Follow‐up, mo, median (IQR)	28 (14‐44)

CRT, Chemoradiotherapy; IQR, terquartile range; RT, radiotherapy; TN, tumor-node stage (Union for International Cancer Control, 8th ed., 2017)

### FDG-PET/CT and image analysis

PET studies were conducted before treatment using a combined PET/CT scanner (Discovery ST Elite 16; GE Healthcare, Chicago, IL, USA). The patients fasted for at least four hours to ensure a serum glucose concentration of < 150-mg/dL before receiving an intravenous injection of 185-MBq/kg FDG (FDGscan; Nihon Medi-Physics, Tokyo, Japan), followed by PET scanning. A diagnostic CT scan for fusion was performed using a standard protocol without intravenous contrast (120-kV; auto mA range, 30–250-mA; noise index, 25; thickness, 3.75-mm; pitch, 1.75; beam collimation, 20-mm).

The FDG-uptake was calculated as the SUV with the following formula: SUV = tissue concentration (Bq/g)/{injection dose (Bq)/body weight (g)}. The SUVmax of the primary tumor was determined by the maximum SUV recorded within the region of interest around the tumor.

### Statistical analysis

The median and interquartile range (IQR) were calculated for continuous variables. The predictive performance of SUVmax was assessed by receiver operating characteristic (ROC) curves and the total area under the curve (AUC). The 95% confidence intervals (CIs) for sensitivity, specificity, and AUC were calculated. The Youden index was used to determine the optimal SUVmax cutoff value for predicting a high risk of distant metastasis.

Univariate and multivariate Cox regression analyses were used to determine whether any clinical or treatment-related variables were predictors of distant metastases. Univariate factors with a *p*-value less than 0.05 were included in the multivariate analysis. The results are expressed as the hazard ratio (HR) with 95% CIs. Differences in overall and distant metastasis-free survival were compared using Kaplan–Meier curves and log-rank tests. For all tests, *p* < 0.05 was considered statistically significant. The analyses were performed using IBM SPSS Statistics 20.0 (IBM, Inc., Armonk, NY).

## Results

### Patient characteristics

A total of 121 patients with HPSCC were examined in this study, including 113 (93.4%) males and 8 (6.6%) females (Table 1). During diagnosis, the median age was 67 years (IQR 61–73). At the initial presentation, the tumors were staged as T3–T4 in 63 (52.1%) patients, N2–N3 in 84 (69.4%) patients, and overall III-IV in 98 (81.0%) patients. The pyriform sinus (n = 88, 72.7%) was the most common subsite of the main tumor. The median pretherapeutic SUVmax was 14.4 (IQR 11.8–19.7) for the whole cohort. Fifty-nine (48.8%) patients underwent primary surgery with or without combined postoperative radiotherapy or chemoradiotherapy, whereas 62 (51.2%) patients received primary radiotherapy or chemoradiotherapy. During the median follow-up of 28 months (IQR 14–44 months), recurrence or metastasis occurred in 59 (48.8%) patients at a median of 21 months (IQR 7–42 months). This recurrence or metastasis occurred in a locoregional site in 28 (23.1%) patients and at a distant site in 31 (25.6%) patients. Three patients displayed recurrence and metastasis in both locoregional and distant sites.

### ROC curve analyses and cutoff values for quantitative measurements of FDG-PET/CT

**Fig. 1:**
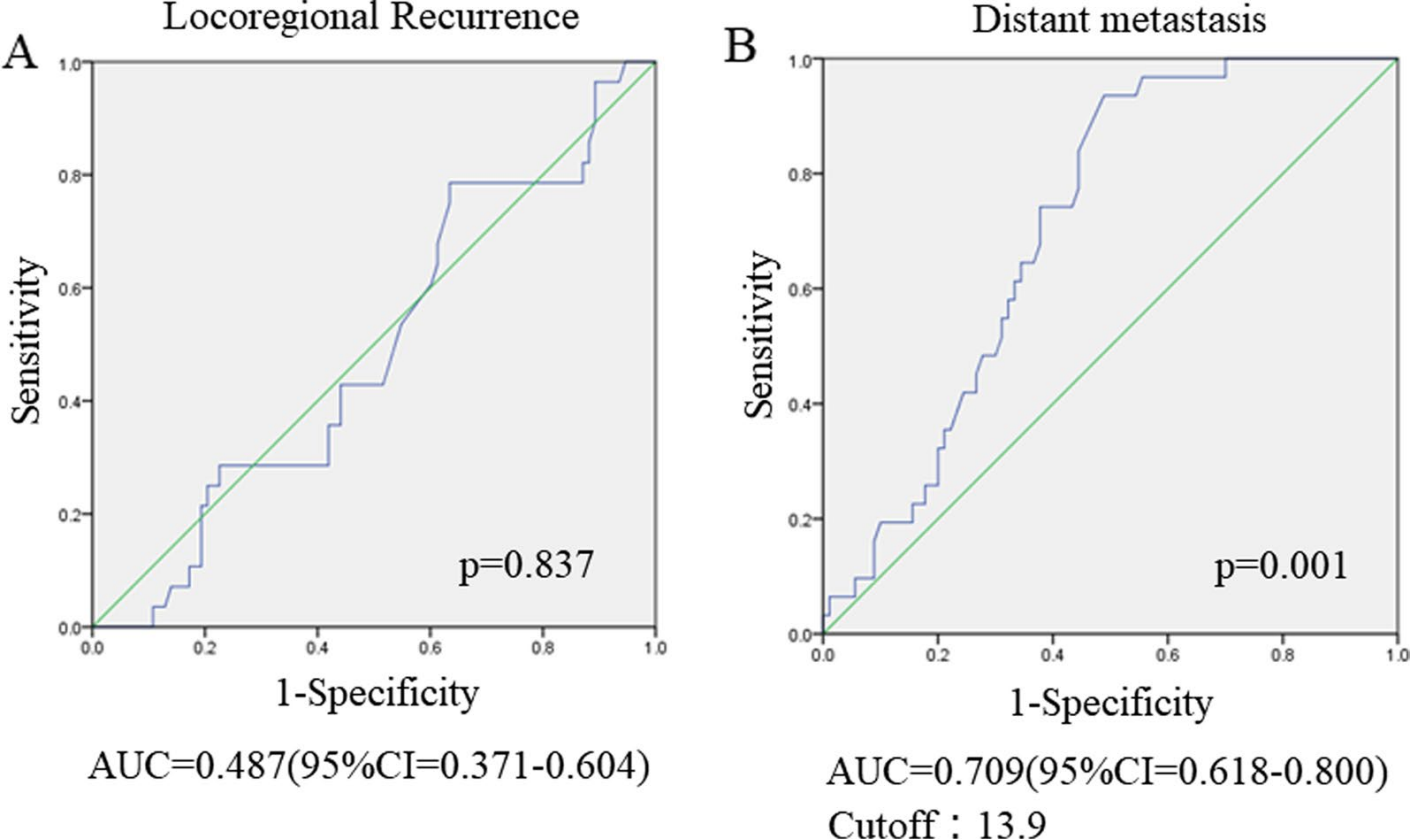
Receiver operating characteristic (ROC) curve analysis of locoregional recurrence (LR) and distant metastasis prediction according to the pretherapeutic SUVmax of the primary tumor. **A** ROC curve for LR showing a lack of significant correlation [area under the curve (AUC) = 0.487] (95% CI = 0.371–0.604, *P* = 0.837). **B** ROC curve for distant metastasis. The area under the ROC curve was 0.709 (95% CI = 0.618–0.800, *P* = 0.001), and 13.9 was determined as the best potential cutoff value based on the Youden index. The sensitivity and specificity for an SUVmax of 13.9 were 93.5% and 51.5%, respectively

In this study, an ROC curve analysis to determine the SUVmax value that was predictive of LR and distant metastasis, was performed, including the AUC analyses. It was found that the pretherapeutic SUVmax was predictive of distant metastasis and that the best potential cutoff value was 13.9 according to the Youden index (AUC = 0.709; 95% CI, 0.618–0.800; *P* = 0.001; sensitivity 93.5%; specificity 51.5%; *P* = 0.001, Fig. 1B). Conversely, the ROC curve for LR did not show a significant correlation (AUC = 0.487; 95% CI, 0.371–0.604; *P* = 0.837) (Fig. 1A).

**Fig. 2:**
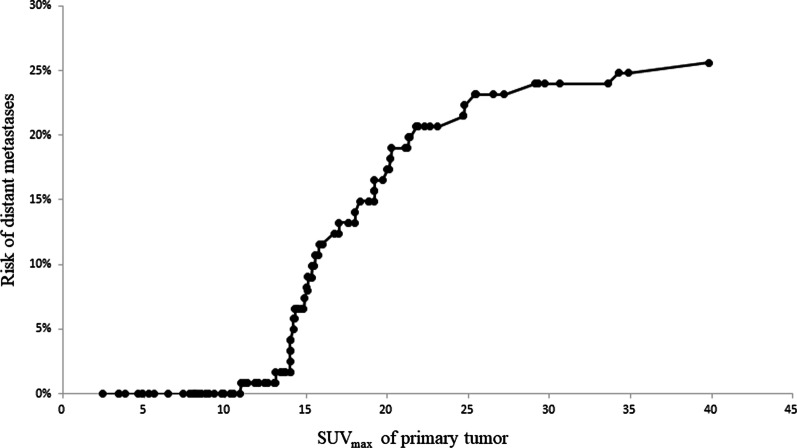
Frequency curve showing the risk of distant metastases according to the SUVmax of the primary tumor arranged in an ordinal fashion. Patients with a low SUVmax (< 13.9) had a low risk of distant metastasis. The risk of distant metastasis increased sharply as the SUVmax increased

According to the pretherapeutic SUVmax of the primary tumor, the risk of distant metastasis was also evaluated in an ordinal fashion. For patients with a low SUVmax (< 13.9), the risk of distant metastasis was low. The risk of distant metastasis increased sharply with a higher SUVmax (≥ 13.9), as shown in Fig. 2.

### Risk factors for distant metastasis and patient survival

Factors possibly predicting distant metastasis were analyzed using univariate and multivariate Cox regression models (Table 2). Univariate analysis showed that T3/4 classification, N2/3 classification, clinical-stage IV, and a pretherapeutic SUVmax ≥ 13.9 were predictors of distant metastasis. The incidence of distant metastasis for these factors is detailed in Table 3. In the multivariate analysis, the only independent predictor of distant metastasis was a pretherapeutic primary tumor SUVmax ≥ 13.9 (HR = 8.61, 95% CI = 1.91–38.8, *P* = 0.005).

**Table 2:** Cox regression analysis for distantmetastasis-free survival for hypopharyngeal cancer patients (No. of patients = 121)

Variables	Univariable analysis			Multivariable analysis		
	HR	95% CI	*P* value	HR	95% CI	*P* value
Gender Male versus female	0.66	0.20–2.17	0.49			
Age ≥ 70 versus < 70 years	1.98	0.98–4.01	0.06			
Primary site PS versus non-PS	1.01	0.45–2.27	0.98			
Treatment Surgery versus radiation	0.85	0.42–1.73	0.66			
T-classification T3 + T4 versus T1 + T2	3.37	1.50–7.55	0.003*	1.52	0.65–3.58	0.33
N-classification N2 + N3 versus N0 + N1	2.96	1.14–7.72	0.026*	1.51	0.20–11.2	0.69
Clinical stage Stage IV versus I + II + III	3.33	1.17–9.54	0.025*	1.06	0.11–9.85	0.96
SUV_max_ primary tumor ≥ 13.9 versus < 13.9	12.09	2.88–50.7	0.001*	8.61	1.91–38.8	0.005*

HR: Hazard ratio. 95% CI: 95% confidence interval. SUV max: maximum standard uptake value. *P* value for null hypothesis; *statistically significant

**Table 3:** Incidence of distant metastasis by TN and clinical stage, and SUVmax

Characteristics	No. of patients (n = 121)	No. of distant metastasis (n = 31)	%
*T-classification*			
T1	8	1	12.5
T2	50	7	14.0
T3	27	9	33.3
T4	36	14	38.9
*N-classification*			
N0	29	3	10.3
N1	10	2	20.0
N2	78	25	32.1
N3	4	1	25.0
*Clinical stage*			
I	6	1	16.7
II	17	1	0.6
III	12	3	25.0
IV	86	26	30.2
*SUV*_*max*_* primary tumor*			
< 13.9	48	2	4.2
≥ 13.9	73	29	39.7

TN, tumor and node stage (Union for International Cancer Control, 8th ed., 2017). SUVmax: maximum standard uptake value

**Fig. 3:**
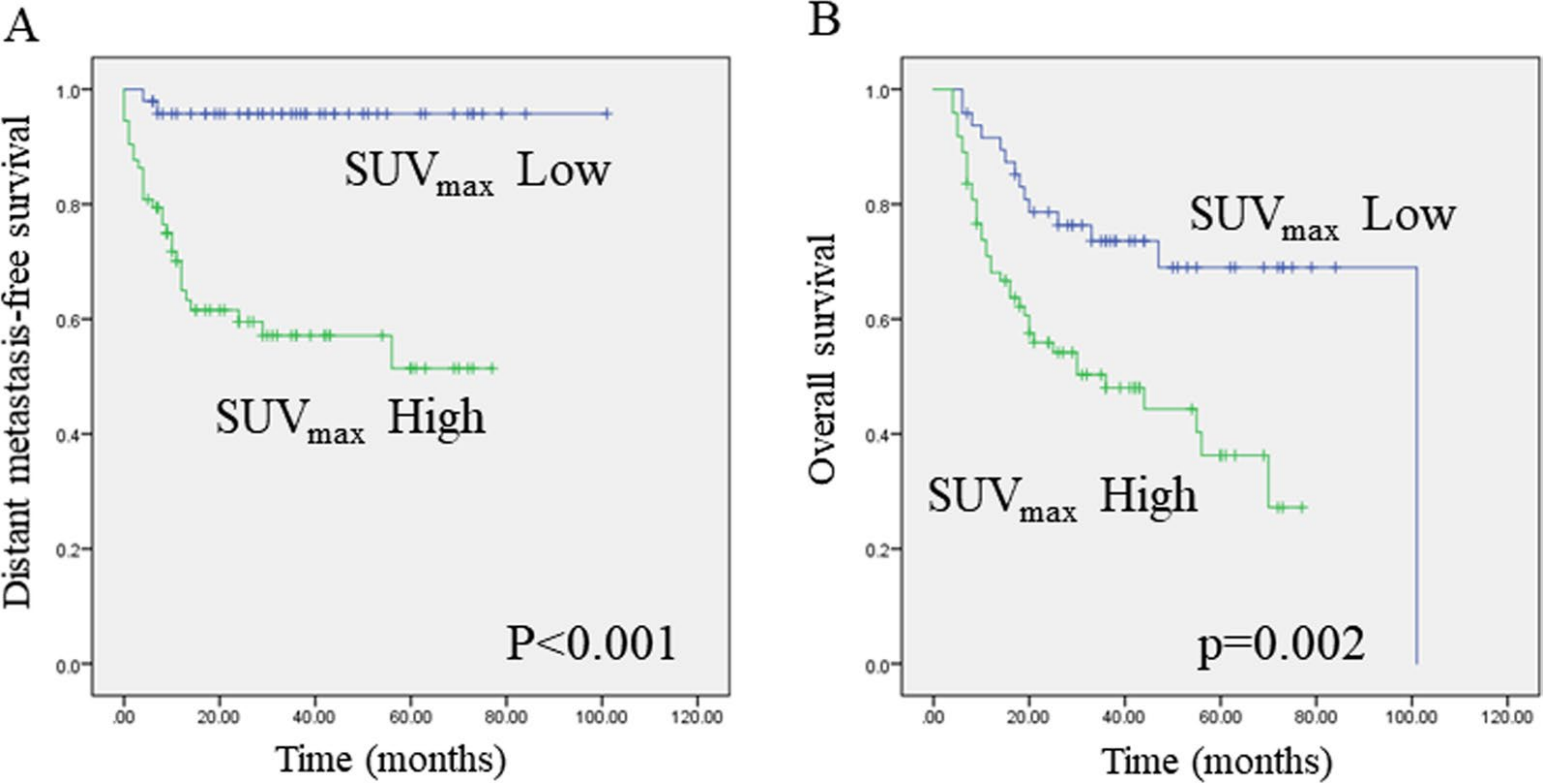
Kaplan–Meier curves showing distant metastasis-free survival or overall survival. **A** A high SUVmax predicted poorer distant metastasis-free survival (**A**) and overall survival (**B**) in hypopharyngeal cancer patients (log-rank test, *P* < 0.001 and *P* = 0.002, respectively)

Additionally, the relationship between pretherapeutic SUVmax and survival outcomes was evaluated. A comparative Kaplan–Meier survival analysis showed a worse distant metastasis-free and overall survival in patients with a pretherapeutic SUVmax ≥ 13.9 (Fig. 3A and B, log-rank test, *P* < 0.001, and *P* = 0.002, respectively).

## Discussion

Distant metastases of head and neck cancers are present in approximately 10% of cases at diagnosis, with additional 20%–30% developing metastases throughout their disease [14]. In addition to this clinically recognized occurrence, the incidence of distant metastases in autopsy series is three to four times higher than that in clinical series [15, 16]. Once distant metastasis occurs in head and neck squamous cell carcinoma (HNSCC), the prognosis is relatively poor (approximately ten months), even with the best treatments related to recent drug developments [17]. Thus, approximately 15–20% of patients with HNSCC die of distant metastasis [16].

HPSCC, in particular, has the most frequent incidence of distant metastasis among HNSCC [14, 16], and distant metastasis has occurred in 60% of patients who died of hypopharyngeal cancer [15]. Thus, distant metastasis is a clinically significant problem directly related to HNSCC prognosis, particularly HPSCC. However, it has long been highlighted that the head and neck cancer prognosis can be improved if distant metastasis can be controlled [18].

Recently, following the development of new drugs, particularly immune checkpoint inhibitors, it has become possible to prolong the survival of patients with distant metastasis [19, 20]. Therefore, predicting distant metastasis and performing appropriate treatment early is becoming increasingly important for improving HNSCC progonisis, particularly HPSCC.

This study examined 121 cases of HPSCC and found that the SUVmax in the primary lesion before treatment was an independent predictor of distant metastases. Notably, it was found that the low SUVmax group had few distant metastases, but metastases incidence increased rapidly above the cutoff value, as shown in Fig. 2. Conversely, following the T and N classifications, distant metastases were scattered, even at a relatively early stage (Table 3). Because of these characteristics, SUVmax may have been the only independent predictor of distant metastasis in multivariate analysis.

A higher uptake of FDG shows active tumor metabolism and correlates negatively with tumor oxygenation [21]. Many studies have revealed that poor tumor oxygenation or hypoxia is related to higher tumorigenicity, resulting in a poor clinical prognosis, including distant metastasis [22]. Alternatively, the SUVmax, which is obtained from FDG accumulation, directly reflects the condition inside the primary tumor. Therefore, SUVmax is thought to better judge the nature of the tumor, which is thought to be why SUVmax was considered an independent index of distant metastasis in this study.

In addition to SUVmax, metabolic tumor parameters derived from FDG-PET include metabolic tumor volume (MTV) and total lesion glycolysis (TLG). Many studies have reported the usefulness of these parameters in head and neck cancer [23, 24]. However, among these metabolic parameters, SUVmax is thought to have an advantage over MTV and TLG because, in clinical practice, MTV and TLG are susceptible to fluctuations induced by differences in the definition of areas of interest and adjacent FDG-avid structures [5, 25]. These factors do not affect SUVmax. Therefore, SUVmax has only small differences among observers, is highly reliable and is easy to introduce compared with MTV and TLG [5, 26]. For these reasons, SUVmax is considered the most suitable metabolic tumor parameter derived from FDG-PET.

This study has several limitations. First, a comparatively small number of patients were obtained from only one institution, and a retrospective design was used. Secondly, the presence or absence of chemotherapy combined with radiation or surgery could not be considered. In addition to these limitations, the study included cases in which radiation therapy and surgical treatment was used as the initial treatment. In locally advanced cases, the primary site, the hypopharynx, may have been resected by surgery. In these cases, it is inevitably difficult to assess local recurrence. Therefore, it is possible that SUVmax was not a predictor of locoregional recurrence in this study.

Further large-scale studies that focus on the initial treatment of the target patients, with a prospective design is necessary.

Conclusively, this study highlights the role of SUVmax in pretreatment primary tumors of HPSCC and shows that SUVmax is significantly associated with distant metastasis, distant metastasis-free survival, and overall survival in a retrospective study.

## Data Availability

The datasets used and analyzed during the current study are available from the corresponding author on reasonable request.

## Abbreviations

AUC: Area under the curve
CT: Computed tomography
FDG-PET: 18-Fluorodeoxyglucose positron emission tomography
HNSCC: Head and neck squamous cell carcinoma
HPSCC: Hypopharyngeal squamous cell carcinoma
ROC: Receiver operating characteristic
SUVmax: Maximum standardized uptake value

## References

[1] Newman JR, Connolly TM, Illing EA, Kilgore ML, Locher JL, Carroll WR (2015). Survival trends in hypopharyngeal cancer: a population-based review. Laryngoscope.

[2] Creff G, Devillers A, Depeursinge A, Palard-Novello X, Acosta O, Jegoux F, Castelli J (2020). Evaluation of the prognostic value of FDG PET/CT parameters for patients with surgically treated head and neck cancer: a systematic review. JAMA Otolaryngol Head Neck Surg.

[3] Li Y, Ou X, Hu C (2019). Prevalence and prognostic impact of synchronous distant metastases in patients with hypopharynx squamous cell carcinomas: a SEER-based study. J Cancer.

[4] Spector GJ (2001). Distant metastases from laryngeal and hypopharyngeal cancer. ORL.

[5] Castelli J, De Bari B, Depeursinge A, Simon A, Devillers A, Roman Jimenez G, Prior J, Ozsahin M, de Crevoisier R, Bourhis J (2016). Overview of the predictive value of quantitative 18 FDG PET in head and neck cancer treated with chemoradiotherapy. Crit Rev Oncol Hematol.

[6] Doi H, Kitajima K, Fukushima K, Kawanaka Y (2016). SUV max on FDG-PET is a predictor of prognosis in patients with maxillary sinus cancer. Jpn J Radiol.

[7] Suzuki H, Kato K, Fujimoto Y, Itoh Y, Hiramatsu M, Maruo T, Naganawa S, Hasegawa Y, Nakashima T (2013). 18F-FDG-PET/CT predicts survival in hypopharyngeal squamous cell carcinoma. Ann Nucl Med.

[8] Werner J, Hüllner MW, Rupp NJ, Huber AM, Broglie MA, Huber GF, Morand GB (2019). Predictive value of pretherapeutic maximum standardized uptake value (Suvmax) in laryngeal and hypopharyngeal cancer. Sci Rep.

[9] Lee JR, Almuhaimid TM, Roh JL, Oh JS, Kim SJ, Kim JS, Choi SH, Nam SY, Kim SY (2018). Prognostic value of 18F-FDG PET/CT parameters in patients who undergo salvage treatments for recurrent squamous cell carcinoma of the larynx and hypopharynx. J Surg Oncol.

[10] Suzuki H, Kato K, Nishio M, Tamaki T, Fujimoto Y, Hiramatsu M, Hanai N, Kodaira T, Itoh Y, Naganawa S (2017). FDG-PET/CT predicts survival and lung metastasis of hypopharyngeal cancer in a multi-institutional retrospective study. Ann Nucl Med.

[11] Brierley JD, Gospodarowicz MK, Wittekind C. TNM classification of malignant tumours, 8th edition | Wiley. Wiley-Blackwell (2017) Available at: https://www.wiley.com/en-us/TNM+Classification+of+Malignant+Tumours,+8th+Edition-p-9781119263579. Accessed January 12, 2022

[12] Ishizuka M, Fujimoto Y, Itoh Y, Kitagawa K, Sano M, Miyagawa Y, Ando A, Hiramatsu M, Hirasawa N, Ishihara S (2011). Relationship between hematotoxicity and serum albumin level in the treatment of head and neck cancers with concurrent chemoradiotherapy using cisplatin. Jpn J Clin Oncol.

[13] Nakahara R, Kodaira T, Furutani K, Tachibana H, Tomita N, Inokuchi H, Mizoguchi N, Goto Y, Ito Y, Naganawa S (2012). Treatment outcomes of definitive chemoradiotherapy for patients with hypopharyngeal cancer. J Radiat Res.

[14] Pisani P, Airoldi M, Allais A, Valletti PA, Battista M, Benazzo M, Briatore R, Cacciola S, Cocuzza S, Colombo A (2020). Metastatic disease in head and neck oncology. Acta Otorhinolaryngol Ital.

[15] Kotwall C, Sako K, Razack MS, Rao U, Bakamjian V, Shedd DP (1987). Metastatic patterns in squamous cell cancer of the head and neck. Am J Surg.

[16] Duprez F, Berwouts D, De Neve W, Bonte K, Boterberg T, Deron P, Huvenne W, Rottey S, Mareel M (2017). Distant metastases in head and neck cancer. Head Neck.

[17] Vermorken JB, Mesia R, Rivera F, Remenar E, Kawecki A, Rottey S, Erfan J, Zabolotnyy D, Kienzer H-R, Cupissol D (2008). Platinum-based chemotherapy plus cetuximab in head and neck cancer. N Engl J Med.

[18] Wiegand S (2016). Survival after distant metastasis in head and neck cancer. Head Neck.

[19] Ferris RL, Blumenschein G, Fayette J, Guigay J, Colevas AD, Licitra L, Harrington K, Kasper S, Vokes EE, Even C (2016). Nivolumab for recurrent squamous-cell carcinoma of the head and neck. N Engl J Med.

[20] Burtness B, Harrington KJ, Greil R, Soulières D, Tahara M, de Castro G, Psyrri A, Basté N, Neupane P, Bratland Å (2019). Pembrolizumab alone or with chemotherapy versus cetuximab with chemotherapy for recurrent or metastatic squamous cell carcinoma of the head and neck (KEYNOTE-048): a randomised, open-label, phase 3 study. Lancet.

[21] Wilson WR, Hay MP (2011). Targeting hypoxia in cancer therapy. Nat Rev Cancer.

[22] Rankin EB, Giaccia AJ (2016). Hypoxic control of metastasis. Science.

[23] Pak K, Cheon GJ, Nam HY, Kim SJ, Kang KW, Chung JK, Kim EE, Lee DS (2014). Prognostic value of metabolic tumor volume and total lesion glycolysis in head and neck cancer: a systematic review and meta-analysis. J Nucl Med.

[24] Park GC, Kim JS, Roh JL, Choi SH, Nam SY, Kim SY (2013). Prognostic value of metabolic tumor volume measured by 18F-FDG PET/CT in advanced-stage squamous cell carcinoma of the larynx and hypopharynx. Ann Oncol.

[25] Krak NC, Boellaard R, Hoekstra OS, Twisk JWR, Hoekstra CJ, Lammertsma AA (2005). Effects of ROI definition and reconstruction method on quantitative outcome and applicability in a response monitoring trial. Eur J Nucl Med Mol Imaging.

[26] Paidpally V, Chirindel A, Lam S, Agrawal N, Quon H, Subramaniam RM (2012). FDG-PET/CT imaging biomarkers in head and neck squamous cell carcinoma. Imaging Med.

